# Case report: A disconjugate pattern in video head impulse testing hints toward a central cause of acute vertigo

**DOI:** 10.3389/fneur.2023.1222475

**Published:** 2023-07-27

**Authors:** Vincent G. Wettstein, Bertram Feil, Marie-Luise Mono

**Affiliations:** ^1^Department of Otolaryngology, Rautipraxis, Zurich, Switzerland; ^2^Department of Radiology, Stadtspital Zürich Triemli, Zurich, Switzerland; ^3^Department of Neurology, Stadtspital Zürich Triemli, Zurich, Switzerland

**Keywords:** binocular, INO, neurosarcoidosis, disconjugate, internuclear ophthalmoplegia, video head impulse test (VHIT)

## Abstract

When acute vertigo occurs, the challenge for the medical practitioner lies in the focused assessment to find the cause of its symptoms. Especially in the case of central pathology, a fast diagnosis is essential for therapy. The **h**ead impulse, **n**ystagmus, **t**est of **s**kew (HINTS) protocol and the additional video head impulse test (VHIT) can distinguish between central and peripheral vestibular causes in the acute setting and thus help to set the right path for further evaluation and treatment. In this case, a patient with acute onset of vertigo presented with an unusual pattern in the VHIT. Binocular eye tracking showed a disconjugate horizontal vestibulo-ocular reflex (VOR) with severe loss or gain for the adducting eye yet with a lack of corrective saccades. The abducting eye produced a pattern of mild VOR gain loss yet with pronounced corrective saccades. Together with clinical findings that were compatible with internuclear ophthalmoplegia, a probable central lesion in the medial longitudinal fasciculus (MLF) region was suspected. The patient was sent to a tertiary hospital, where the initial MRI was negative, but due to additional neurological symptoms occurring later, multiple lesions in the cervical spine and cerebellum were detected. The hypothesis of an inflammatory demyelinating disease of the central nervous system (CNS) was made. A further workup led to the final diagnosis of neurosarcoidosis. In a retrospective neuroradiologic assessment, an alteration compatible with a non-active demyelinating lesion in the MLF was detected on secondary imaging as a probable cause of the initial pathophysiologic finding. In this report, we aimed to highlight the unusual case of a disconjugate VOR as a distinctive VHIT pattern hinting toward a central cause of acute vertigo that clinicians should be aware of.

## 1. Introduction

The assessment of a patient with acute vertigo in order to come to appropriate conclusions for treatment is challenging. A variety of causes can lead to dizziness and vertigo. While peripheral-vestibular disorders account for the majority of cases (1), acute neurologic pathologies, such as stroke, can also present with dizziness without any other leading symptoms (2). Over the last few years, focused clinical examination guidelines were established in order to quickly reach a conclusive diagnosis for further treatment. The **h**ead impulse, **n**ystagmus, **t**est of **s**kew (HINTS) protocol enables clinicians to distinguish between central and peripheral disorders with high precision (3). Additionally, the video head impulse test (VHIT) serves as a fast tool to measure semicircular canal function (4–6) and can therefore contribute to the first assessment.

However, in some cases, we are unable to achieve diagnosis in the acute setting due to challenging clinical examination when symptoms are severe and cooperation is reduced or due to unclear or unexpected clinical findings.

In this report, we present a case where we were challenged by an unusual VHIT finding not yet described in a patient with acute vertigo. A subsequent workup led to the diagnosis of a rare neurological disease.

## 2. Case description

A 55-year-old patient presented with acute vertigo. He complained about aggravated symptoms with movements of the head and an unstable gait, as well as nausea and vomiting. Apart from treatment for hypertension and hypercholesterinemia, the patient was healthy.

**Figure 1:**
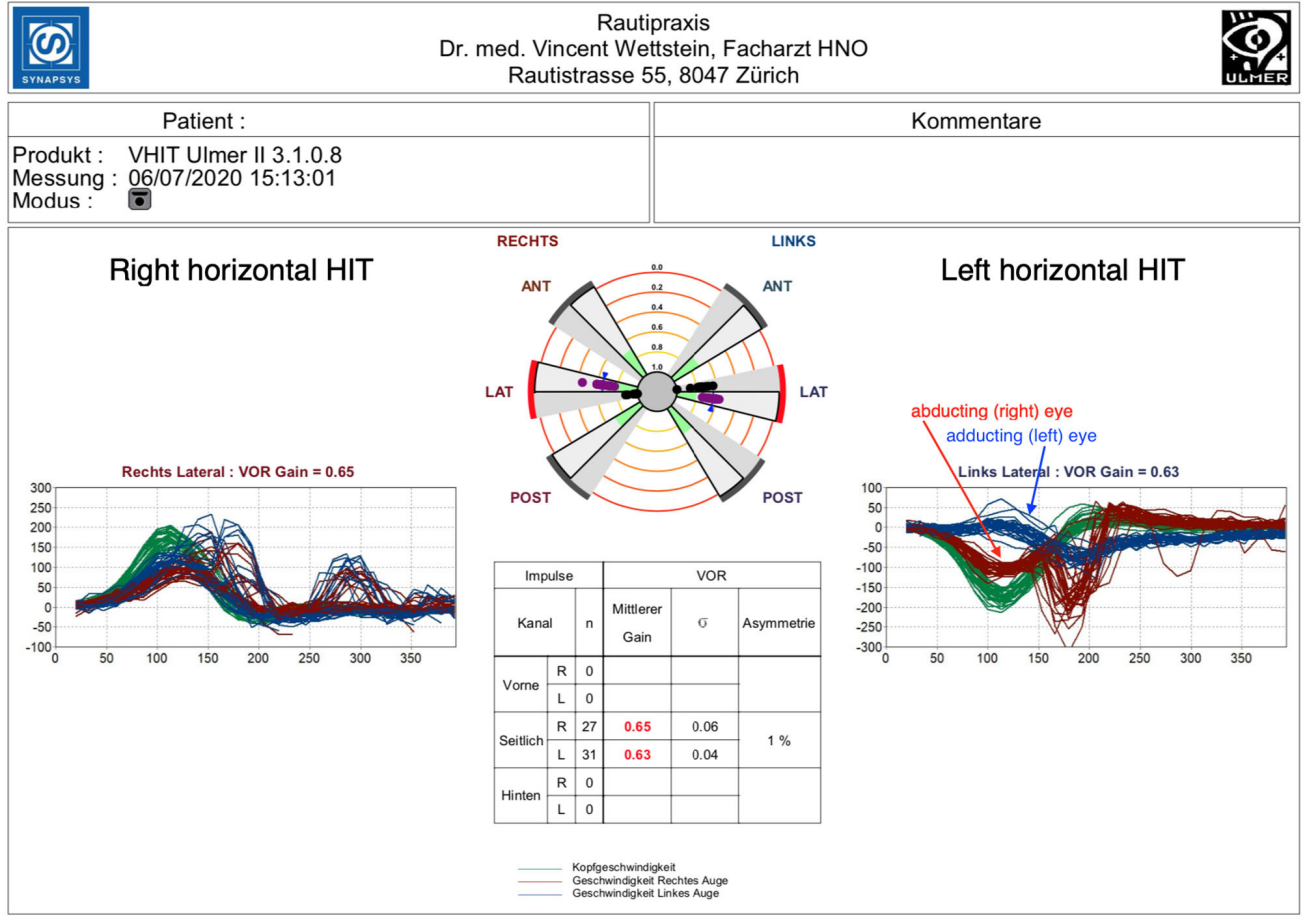
Right horizontal HIT: mild hypofunction with a reduced gain of 0.65 (normal value = >0.8) and both covert and overt saccades. Left horizontal HIT: disconjugate pattern. Mild to moderate gain reduction of 0.61 with a robust covert saccade bundle of the right abducting eye (red). Left adducting eye analysis (blue) shows a severe gain reduction of 0.09 yet no correction saccades.

Clinical findings were as follows: predominantly down beating nystagmus in eccentric gaze positions, with and without fixation. With attempted right gaze, an adduction paresis of the left eye and an abduction nystagmus of the right eye were documented, consistent with the clinical phenotype of left internuclear ophthalmoplegia. With left gaze, no gaze palsy or abduction nystagmus was seen. Vertical eye movements were normal. The test of skew showed no ocular tilt reaction. The clinical head impulse test was inconclusive; therefore, binocular video head impulse testing [Synapsys VHIT Ulmer 2.0, Marseille (7)] was performed (Figure 1).

Horizontal semicircular canal testing with binocular eye tracking showed a reduced gain on both sides (0.65 on the right and 0.63 on the left, normal value = >0.8) and an asymmetry ratio of 1%.

The cumulative impulse graph on the right side was congruent with a mild loss of horizontal semicircular canal function, showing slightly reduced gain and both covert and overt saccades (8). Individual gain analysis revealed a higher gain (0.67) for the left (abducting) eye than for the right (adducting) eye (0.48), yet covert and overt corrective saccades were detected for both eyes in a similar pattern.

Head impulses to the left side, however, showed a disconjugate pattern of the abducting and adducting eye. Measurement of the right (abducting) eye revealed a mild to moderate gain reduction of 0.61 with a robust saccade bundle starting ~150–200 ms after the initiation of the head impulse. Left (adducting) eye analysis showed a severe gain reduction of 0.09 and a slight elevation of eye velocity toward the end of the head movement, yet there were no correction saccades following the head impulse in spite of the severe loss of gain.

Applying the HINTS protocol to these findings, VOR gain reduction and corrective saccades should suggest a loss of canal function and therefore a peripheral cause, while downbeating gaze nystagmus, as well as gaze palsy, hint toward central disorders. The disconjugate VHIT pattern of the right and left eye could not be attributed to a peripheral-vestibular disorder but was rather associated with a probable central lesion in the medial longitudinal fasciculus (MLF) region, as described in a scleral search coil study by Aw et al. (9).

## 3. Diagnostic assessment, treatment, follow-up, and outcomes

**Figure 2:**
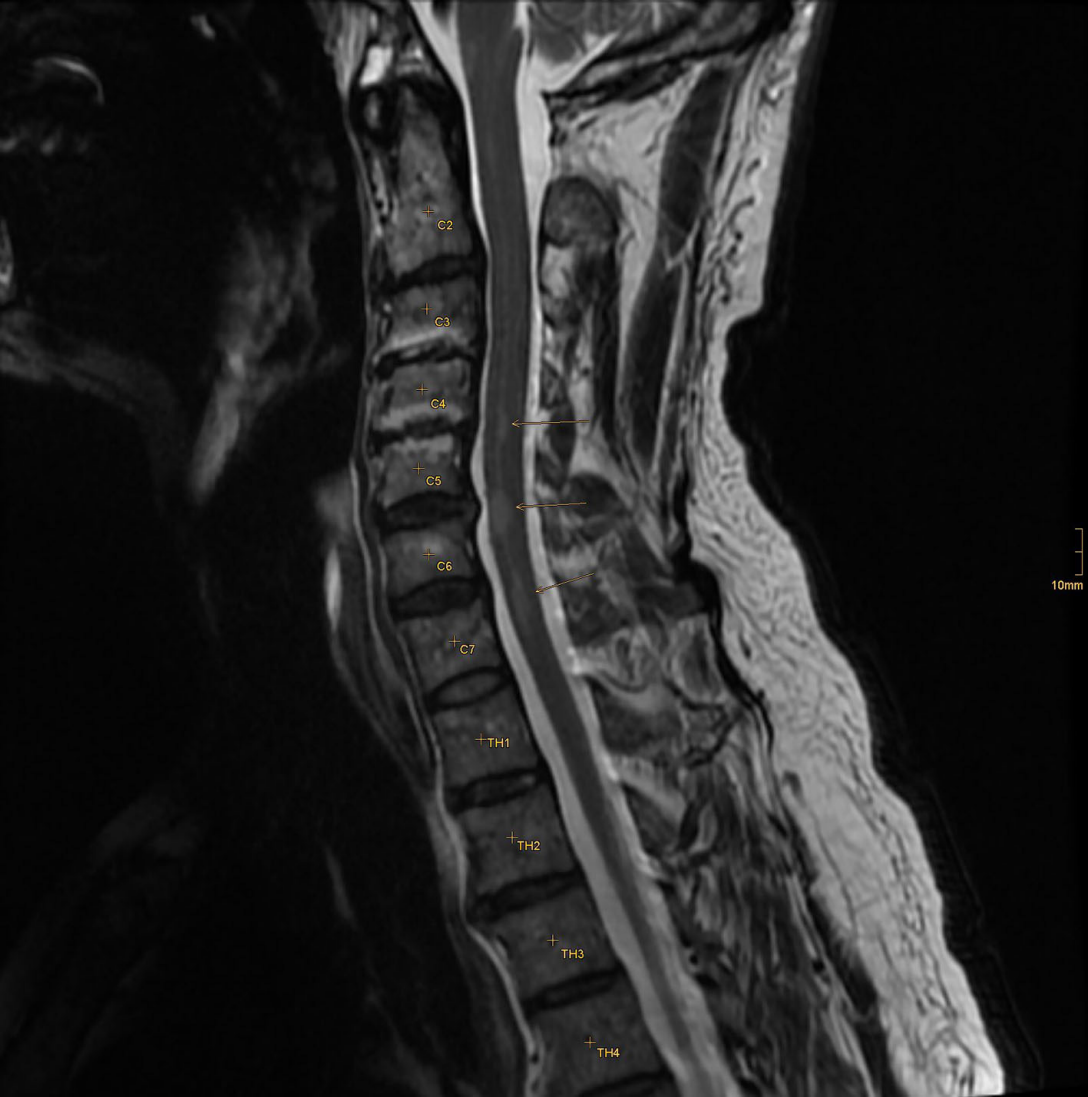
Three T2W hyperintense lesions in the cervical myelon.

**Figure 3:**
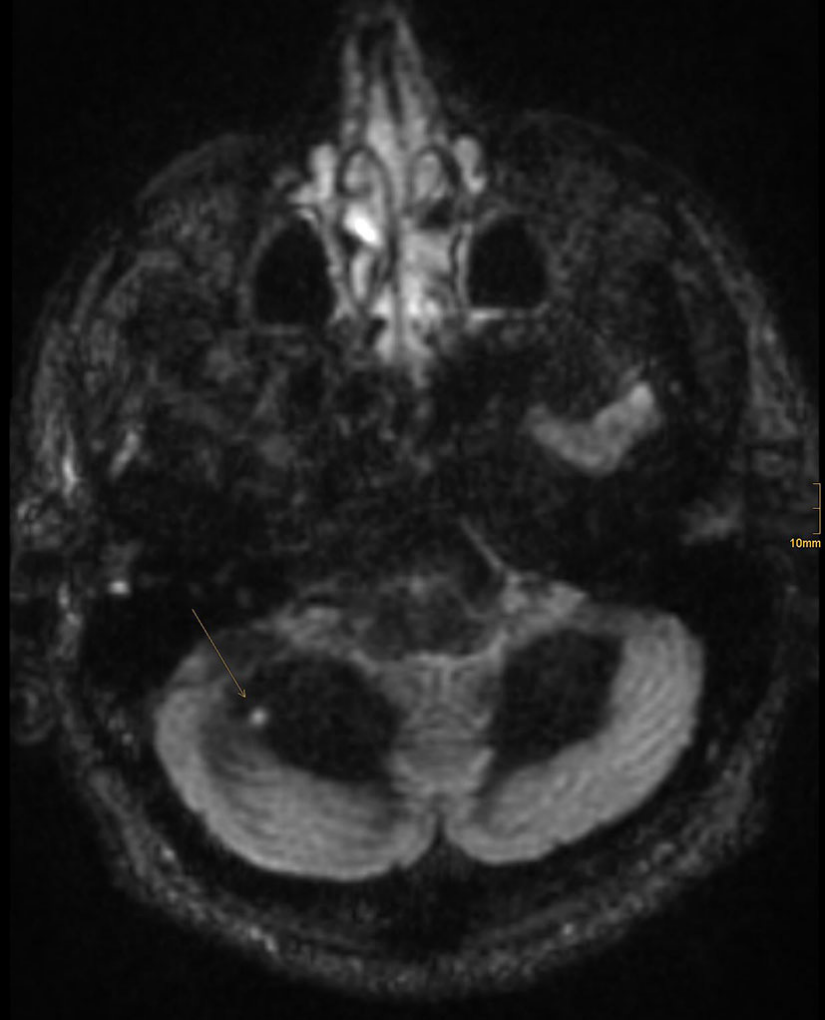
One FLAIR hyperintense lesion in the right cerebellar hemisphere showing contrast enhancement.

The patient was immediately admitted to the neurologic department of a tertiary hospital for further diagnosis and treatment. An MRI of the brain showed no recent ischemic or inflammatory lesion but some non-specific white matter lesions. An MRI-negative stroke due to small vessel disease was suspected, and treatment with aspirin and clopidogrel was started. A further stroke workup showed no other etiology, and the patient was discharged to home. A few days later, the patient was re-admitted due to progressive dizziness and paresis of the right leg. Extended MR imaging revealed three inflammatory lesions in the cervical spine and a hyperintense lesion in the right cerebellar hemisphere showing contrast enhancement (Figures 2, 3).

The cerebrospinal fluid (CSF) on the lumbar puncture showed pleocytosis in 86 cells and slightly elevated protein and lactate levels. There was an intrathecal IgG synthesis combined with a systemic immune reaction. There were no signs of an infective disease or systemic vasculitis. A chronic remitting inflammatory demyelinating disease of the central nervous system (CNS), e.g., multiple sclerosis, was suspected.

The patient was treated with high-dose intravenous (IV) prednisolone for 5 days and transferred to a rehabilitation clinic where his condition initially improved.

Because of a prolonged stay at the rehabilitation clinic, the follow-up examination at the tertiary hospital was several weeks later than initially planned. The patient, then, reported progressive weakness in the right leg and paresthesia in both legs. Clinical examination revealed an impaired monoparesis of the right leg, a severe disturbance of proprioception of the leg left, and a sensitive disturbance below the eighth thoracic vertebra (T8) on both sides due to new inflammatory lesions in the cervical and thoracic spinal cord. CSF puncture revealed a mononuclear pleocytosis of 62 cells and elevated protein levels. The aquaporin and myelin oligodendrocyte glycoprotein (MOG) antibodies in serum and CSF were negative. There were also no clinical or laboratory signs of an underlying systemic disease such as systemic lupus erythematosus (SLE) or any systemic vasculitis. The sIL2 receptor in the CSF was elevated (365.1). Together with the intrathecal IgG synthesis, combined with signs of a systemic immune reaction and pleocytosis > 50, without evidence for any infective or any other autoimmune disease, this indicated the diagnosis of neurosarcoidosis.

The patient was again treated with high-dose IV prednisolone followed by oral tapering and rituximab (initially 2 × 1,000 mg). He continued to receive this treatment every 6 months with 1,000 mg. Since then, his medical condition has been stable without any evidence of disease activity or affection of the lung.

## 4. Discussion

To the best of our knowledge, this is the first description of a VHIT finding with disconjugate horizontal VOR in patients with acute vertigo. We report this case to highlight the correlation between the clinical finding of acute INO and a distinctive, disconjugate VHIT pattern. It reveals severe VOR gain reduction yet missing corrective saccades in the head impulse analysis of the adducting eye, whereas the results for the abducting eye display a pattern consistent with mild semicircular canal hypofunction and corrective saccades.

Disconjugate VOR deficits have been shown in multiple sclerosis patients with internuclear ophthalmoplegia (INO) by Aw et al. (9) using the binocular scleral search coil technique. In this study, the same distinctive pattern of VOR disconjugacy was found for both unilateral and bilateral INO. The adducting eye revealed a more severe loss of VOR, but no corrective saccades, whereas the abducting eye had larger VOR gains and corrective saccades. Since the same pattern was found in our case, a central-vestibular disorder was suspected and ultimately diagnosed in the form of a chronic remitting inflammatory demyelinating disease. We can, therefore, confirm a disconjugate VOR deficit originating from a CNS disease by using the binocular VHIT technique. Recently, Grove et al. were able to show that multiple sclerosis (MS) patients with low VOR gain have reduced or absent compensatory saccades (CSs) (10, 11). They further postulated that the lack of CS in correlation with reduced VOR in VHIT may be pathognomonic for INO. Using binocular VHIT assessment, we were able to specify this pattern to the adducting eye in INO. Together with the higher gain of the abducting eye in combination with present CS, we argue that this pattern could be a pathognomonic (binocular) VHIT sign for INO.

Another study using monocular VHIT on patients with INO found a deficit of the contralateral posterior canal (12). Since only one eye was tracked, this finding is of importance for clinicians using monocular VHIT systems. However, we argue that by using monocular VHIT equipment, VOR disconjugacy in correlation with clinical INO would have been missed, and therefore, the diagnosis could not have been made.

**Figure 4:**
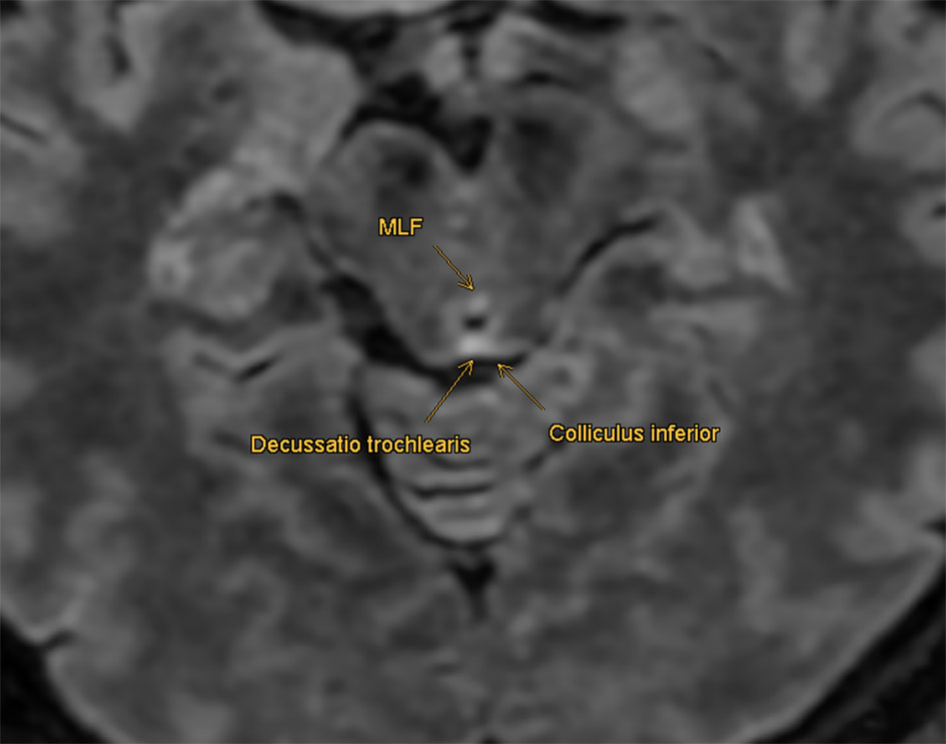
Retrospective imaging analysis: MLF shows an enhanced signal in the FLAIR protocol at the level of the inferior colliculi, compatible with a non-active demyelinating lesion. No correlation in the diffusion-weighted sequences and no enhancement with contrast agents were found retrospectively.

The MRI following the first and second assessments revealed remitting inflammatory lesions in the spinal cord and the cerebellum, with additional neurological workup finally leading to the diagnosis of neurosarcoidosis. With the initial clinical findings described above, we suspected a lesion in the medial longitudinal fasciculus (MLF). However, primary imaging did not reveal pathology in this region. In a retrospective neuroradiological assessment, we were able to discover a small lesion in probable correlation with the patient's neurologic visual disorder in the secondary MRI imaging. At the level of the inferior colliculi, the MLF showed an enhanced signal in the FLAIR protocol (Figure 4).

The small finding had no correlation in the diffusion-weighted sequences and no enhancement with contrast agents. Nevertheless, it is compatible with a non-active demyelinating lesion that could have caused the initial pathophysiological alteration. Why this MLF lesion was not detected at the time of primary imaging remains unclear. An explanation could be the short-time interval between the onset of the clinical finding and the first MRI. Small lesions are known to be missed in acute dizzy patients (3, 13). Inadequate slice thickness could have also led to a missed lesion in the MLF region.

## 5. Patient perspective

In conclusion, our case supports the use of VHIT assessment in patients with acute dizziness or vertigo. By using bilateral eye tracking, ocular disconjugate VOR deficits can be revealed, leading to an accelerated and focused investigation of central pathologies. In this case, we report a distinctive VHIT pattern in correlation with the clinical finding of acute internuclear ophthalmoplegia. In the case of this patient, the origin of the pathological finding was only discovered in the further course of the disease. With our report, we hope to facilitate vestibular assessment for clinicians dealing with similar cases with a specific diagnostic feature. This might be especially helpful in patients with clinically occult INO or reduced cooperation. Further reports of acute cases with analogical binocular VHIT findings may support our hypothesis. Since new VHIT studies using binocular eye tracking emerge (14) and novel devices enter the VHIT market, we are confident that our findings will be of value in future diagnostics. We, therefore, encourage and recommend the use of VHIT in the emergency setting, while highlighting a distinctive finding that clinicians should be aware of.

## Data availability statement

The original contributions presented in the study are included in the article/supplementary material, further inquiries can be directed to the corresponding author.

## Ethics statement

Ethical review and approval was not required for the study on human participants in accordance with the local legislation and institutional requirements. Written informed consent from the patients/participants or patients/participants legal guardian/next of kin was not required to participate in this study in accordance with the national legislation and the institutional requirements. Written informed consent was obtained from the patient for the publication of any potentially identifiable images or data included in this article.

## Author contributions

VGW carried out the first assessment of the patient including the VHIT. He is responsible for the idea, the concept, and the design of the article. MLM led the patient's further follow up and treatment. She contributed substantially to the content of the manuscript. BF investigated on and reviewed the radiologic findings and critically revised the manuscript. All authors contributed to the article and approved the submitted version.
